# Evaluating the effect of an Iranian traditional medicine-based herbal candy on body composition and appetite in overweight and obese adults: A preliminary study

**DOI:** 10.22038/AJP.2022.21314

**Published:** 2023

**Authors:** Maryam Golzar, Effat Saghi, Hassan Rakhshandeh, Zahra Dehnavi, Ali Jafarzadeh Esfehani, Mohsen Nematy

**Affiliations:** 1 *Department of Nutrition, Faculty of Medicine, Mashhad University of Medical Sciences, Mashhad, Iran *; 2 *Pharmacological Research Center of Medicinal Plants, Mashhad University of Medical Sciences, Mashhad, Iran *; 3 *Metabolic Syndrome Research Center, Faculty of Medicine, Mashhad University of Medical Sciences, Mashhad, Iran*

**Keywords:** Iranian traditional medicine, Overweight, Obesity, Weight reduction, Herbal medicine, Herbal candy

## Abstract

**Objective:**

Obesity is an important public health concern in the world. Considering the limited medications and their side effects and lack of a known effective way to reduce appetite, traditional herbs have been considered a complementary treatment for obesity. Therefore, this study was conducted to evaluate the effect of an Iranian Traditional Medicine (ITM)-based herbal candy on body composition and appetite in obese and overweight adults.

**Materials and Methods:**

In this preliminary study that conducted in nutrition clinic of Ghaem Hospital of Mashhad, participants were selected from overweight and obese people and randomly assigned to either groups. Participants in the intervention group received herbal candy which contained a combination of *Portulaca* *oleracea*, *Plantago* *psyllium*, and peanut oil, while participants in the control group received placebo candy for 8 weeks. The primary (appetite response, and weight changes) and secondary (body mass index (BMI), anthropometric parameters, blood pressure, and laboratory variables) outcomes were collected at baseline and during the intervention.

**Results:**

Fifty participants between the age of 18 and 65 years old were included in this study. Herbal candy resulted in a greater reduction in mean weight and BMI compared to placebo (p<0.001). Mean of hunger, satiety, and eating capacity indicators decreased more significantly at all three intervals (30 min after herbal candy, 1 hour after meal and 2 hours after meal) at lunch and dinner meal in the intervention group compared to the control group (p<0.05).

**Conclusion:**

A combination of produced herbal candy at a dose of 4 g (2 pcs), given half an hour before each meal for 8 weeks, can be effective in reducing weight and appetite in obese and overweight people.

## Introduction

The global epidemic of obesity is one of the main concerns of public health, which has currently affected more than 2 billion people all over the world (Swinburn et al., 2011; Brum et al., 2016). It is expected that one out of five adults will be obese by 2025 (Mohammed et al., 2018). The Framingham cohort study has shown that the risk of death was directly related to the number of years a person has been overweight or obese (Asnawi et al., 2011). Obesity is also a major health problem in Iran. Approximately 70% (385,000) of all recorded mortalities in 2002 in Iran were attributed to chronic diseases, among which the most important were overweight and obesity (Behan et al., 2010). 

Obesity is mainly the result of an imbalance between consumed and expanded energy. This imbalance is due to the current lifestyle characterized by low physical activity and high consumption of high-fat and high-carbohydrate foods (Mohammed et al., 2018). The first step in the standard treatment of obesity is lifestyle modification, and other treatments including medications and surgeries are in the second line of obesity treatment which can be beneficial for some obese individuals. Considering the limitations and complications of these therapies, many people have turned to herbal remedies to reduce appetite and weight. 

For many years, medicinal herbs have been used for medical purposes. Knowing new herbal medicines can help create new and useful herbal remedies. Based on the foundations of traditional medicine, loosening of the cardia valve (the valve between the stomach and the esophagus), decreases the appetite for food. Humidity and oil can loosen the valve and thus reduce the desire for food and appetite (Gorjani, 1976). For this reason, we used ingredients containing mucilage, including *Plantago* *psyllium* which absorbs and gradually releases water, along with peanut oil in the process of making herbal candy. So far, studies have examined the effect of peanuts on appetite and food intake. It reduces appetite through its effects on the nervous system and hormonal control (Reis et al., 2013) due to its high unsaturated fat, fiber, and protein content (Neves Ribeiro et al., 2013). 

Plantago ovata is another herb that has been suggested as a potential herb for controlling appetite. that is a genus of the Plantaginaceae family and it is known as blond psyllium (Sahebkar-Khorasani et al., 2019). Plantago ovata contains a kind of water-soluble fiber that can cause satiation by delaying the degradation and nutrient absorption in the small intestine. It also affects gut hormones and the central nervous system (Brum et al., 2016).

In Persia, the leaves and seeds of *Portulaca oleracea* L. (PA), which is known as ‘*Khorfeh*’, are widely used in cooking and confectionery. PA is also an important medicinal plant in Iranian Traditional Medicine (ITM). In his Canon of Medicine, Ibn Sina (981–1037), a well-known Iranian philosopher and physician, recommended PA for the treatment of severe inflammations, erysipelas, pulsatile headaches caused by hot temperament, eye pain, hemoptysis, gastritis, liver inflammation, and intestinal ulcers. He used PA for the treatment of kidneys and bladder pains and ulcers (Ibn Sina, 2007). Gorjani (1042–1136), another eminent ITM scholar, used PA to treat a broad array of diseases including hematemesis, fevers, insomnia, blepharitis, mouth ulcers, cough, tonsillitis and asphyxia, and nocturnal emissions. He also believed that PA is an effective remedy for heart weakness and palpitation (Gorjani, 1976). Razi (854-925), a distinguished Persian physician, recommended chewing PA leaves for the treatment of teeth sensitivity. He also used the plant externally to cure warts, aphthae, and bleeding (Razi, 1998). Moreover, PA has been reported to be a gastrointestinal tonic, anti-appetite, anaphrodisiac, burn healer, and anti-hemorrhoid (Khorasani, 1992).

Modern investigations have assessed various properties of PA. An animal study concluded that PA seeds can reduce weight and body mass index (BMI) in diabetic rats (Zhou et al., 2015). In another study, administration of PA seeds powder (5 g twice daily) increased albumin and high-density lipoprotein, while serum total cholesterol, triglyceride, low-density lipoprotein, fasting glucose, insulin, body weight, and BMI decreased in diabetics ( El-Sayed , 2011).

Considering the limited medications available for obesity and their side effects and that no effective way has been reported to reduce appetite, this study was conducted to investigate the effect of a combination of herbal compounds, including peanut oil*, Portulaca* *oleracea* L., and Plantago ovata in the form of a herbal candy on appetite and weight loss.

## Materials and Methods

**Study population**

The present study was a double-blind, randomized controlled clinical trial. A random number table was used for participant allocation. The placebo served as a reference drug for comparison. From September 2016 to June 2017, a total of 50 overweight and obese participants aged 18-65 years were recruited from the individuals who were referred to the Nutrition Clinic of Ghaem Hospital, Mashhad, Iran (Figure 1). Overweight /obesity was defined as BMI≥25 kg/m^2^. Patients with allergy to the administered herbs, those who used herbal supplements, those with a history of adherence to weight loss diet in the past 6 months, those with an chronic illness (cardiovascular, pulmonary, kidney, and cancer), and pregnant and lactating women were excluded from the study. All the participants provided details of their demographic characteristics, and medical and medication history.

The protocol of this study was approved by the Ethics Committee of the Mashhad University of Medical Sciences (MUMS) (Ethical Code: IR.MUMS.REC.1393.753). The study protocol is available on the Iranian Ministry of Health website (www.irct.ir) under the code: IRCT 2016062628643 N1. All participants gave written informed consent before participating in the study.

**Figure:**
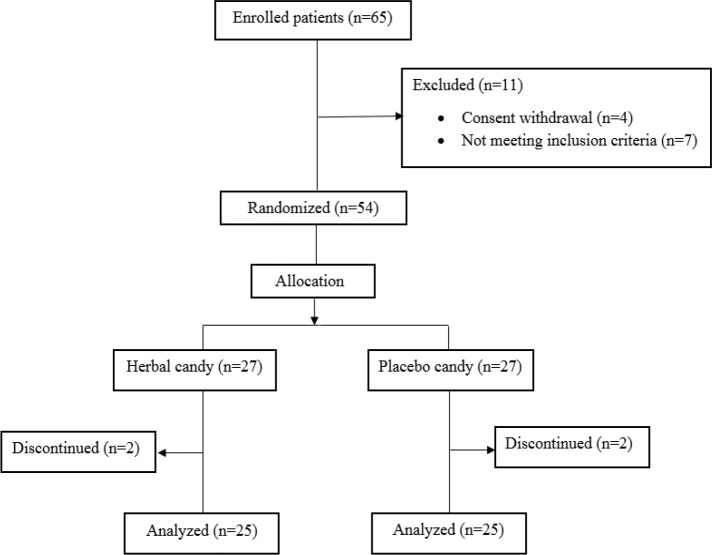
Enrollment of the study

**Randomization**

Randomization was performed by the blocking method. Patients were randomly assigned to either candy and placebo groups. During the project, none of the participants, therapists, or colleagues in project were aware of the type of intervention (medication or placebo). To carry out this research in a double-blind manner, the drug packages were coded as A and B by a person other than the researchers so that the researchers did not know the type of drug received by each group.

**Intervention**

The intervention group received herbal candies, whereas, participants in the control group were given placebo candies with the same shape and color. The herbal candy consisted of *Portulaca* *oleracea* leaves (13%), *Plantago ovata* husks (6%), and peanut oil (9%). The powdered extract of these plants was mixed with peanut oil and excipient containing glucose and sugar (72%) and prepared in the form of a candy weighing 2 g. Placebo was made of 100 percent excipient (Figure 2). In each group, 2 candies (each candy weighed 2 g) were administered half an hour before each main meal (breakfast, lunch, and dinner). All patients were given a low-calorie diet (500 Kcal deficit per day) and dietary recommendations during the study. Subjects’ follow up was done every two weeks and candy consumption was controlled by questioning and counting the cans. The study duration was 8 weeks. Follow-ups were conducted through telephone. 

**Figure:**
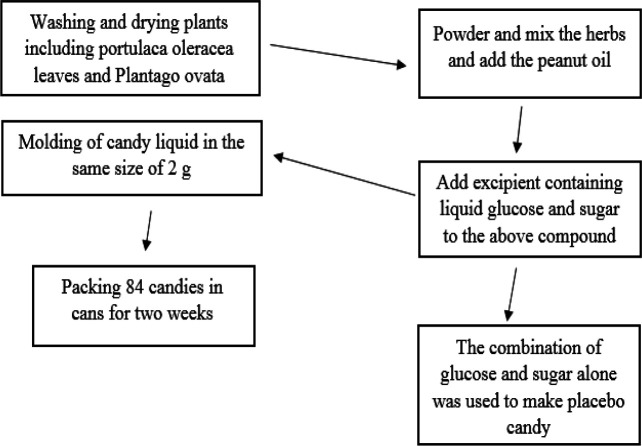
Steps of preparing herbal candy and placebo

**Outcome measures**

The primary outcomes in this study were appetite response and weight changes. Visual Analogue Scale (VAS) was used to assess subjective appetite sensations. The VAS is a validated questionnaire that is designed as a 100 mm horizontal line with words anchored at each end of the line, expressing the most negative and positive rates of satiety, fullness, hunger, and desire to eat. The VAS was filled out by all patients three times a week. The timing of the VAS assessment was before taking candies, and 30 min, 1 hr, and 2 hrs after each meal. Weight was measured by a clinical scale (SECA) to the nearest 0.1 kg. The secondary outcomes were other variables including BMI, anthropometric parameters (height, waist circumference, and hip circumference), blood pressure, and laboratory variables. BMI was calculated as weight in kilograms divided by the squared height in meters (kg/m^2^). Height was measured to the nearest 0.01 cm by using a standard stadiometer without shoes, in a free-standing position. Waist circumference was measured halfway between the lower border of the ribs and the iliac crest in a horizontal plane. Hip circumference was measured at the largest circumference of the pelvis. Systolic and diastolic blood pressure was measured after a 15-20-min rest, with a fitted cuff in the sitting position. Fasting blood samples were collected from the antecubital vein both at the beginning and the end of the study to assess biochemical parameters. The concentration of serum glucose, triglycerides (TG), total cholesterol (TC), high-density lipoprotein-cholesterol (HDL-C), and low-density lipoprotein-cholesterol (LDL-C) were determined by using a commercial kit (Parsazmoon, Tehran, Iran) and Hitachi multi-analyser (Hitachinaka, Japan) following the manufacturer’s instructions. Fasting insulin concentration was obtained by using an enzyme-linked immunosorbent assay (ELISA) commercial kit (Monobind, California, USA). Fasting blood sugar (FBS) and fasting insulin levels were used to calculate HOMA-IR using the following equation: HOMA-IR = glucose (mg/dl) *fasting insulin (μU/ml)/405.

**Statistical analysis**

Data analysis was performed by the statistical package for social sciences (SPSS) software version 24. Descriptive statistics were used to define baseline characteristics. The obtained results were expressed as mean and standard deviations for quantitative data, and the qualitative data are expressed as frequency and percentage. Independent sample t/ Mann-Whitney tests were used to compare quantitative variables between the two groups. Paired-Sample t-test and one-way analysis of variance (ANOVA) tests were used to compare changes between groups. Repeated measures ANOVA was used for VAS questionnaire data. In all the statistical analyses, a p-value of less than 0.05 was considered significant.

## Results

Of the 50 patients, 40 (80%) were female and 10 (20%) were male, with an average age of 37.9±8.3 years and an average BMI of 32.2±4.5 kg/m^2^.

As shown in Table 1, there were no significant differences between the two groups in age, gender proportion, anthropometric parameters, blood pressure, physical activity, or laboratory parameters related to lipid profile and glucose metabolism (p>0.05). 

**Effects of the herbal candy on anthropometric and biochemical parameters**

As Table 2 shows, there was a significant change in the mean weight, BMI, waist circumference, and hip circumference after intervention in both groups (p<0.001). In the intervention group, there was a significant change in mean body fat percentage and waist-hip ratio after the intervention (p=0.01 and p<0.001, respectively), but such difference was not observed in the control group. There was a significant difference in weight, BMI, and body fat percentage between the two groups (p=0.001 and p<0.001, respectively). After the intervention, there was no significant difference in changes in the biochemical parameters, including FBS, TC, HDL-C, LDL-C, TG, fasting insulin, or HOMA-IR, between the two groups. After intervention, serum TG (p=0.01), fasting insulin (p=0.03) and HOMA-IR (p=0.03) significantly decreased only in the intervention group.

**Table:** Clinical and laboratory characteristics of participants at baseline

Variables	Intervention (n=25)	Placebo (n=25)	p-value
Age (years) Height (cm) Weight (kg) BMI (kg/m^2^) Total body fat % FFM (kg) WC (cm) HC (cm) WHR SBP (mmHg) DBP (mmHg) PA (MET-mins/wk) BMR TG (mg/dl) TC (mg/dl) LDL-c (mg/dl) HDL-c (mg/dl) FBS (mg/dl) Fasting insulin (µIU/ml) HOMA-IR	40.5±9 159.3±7.5 79.8±13.9 31.3±3.8 37±5.9 50.3±10.4 96.8±11.6 111.6±8.4 0.86±0.07 127.4±19.7 80.9±14.9 3201.6±2943.7 1678.1±468.9 121.6±38 181.3±44.2 95.1±31.7 45.4±7.2 92.4±14.4 12±9 2.7±2.1	35.6±7.5 160.2±7.9 83.7±15.3 32.5±4.5 38.8±6.2 51.1±10.7 100.3±12.8 114.6±9.4 0.87±0.06 121.9±10.4 78.6±9.7 3369±3036.6 1854.9±430.1 128.6±50.1 190.9±39 94.5±26 42.6±5.2 88.3±8 10.9±6.3 2.4±1.5	0.06 0.68 0.61 0.30 0.28 0.77 0.31 0.23 0.75 0.22 0.52 0.84 0.18 0.58 0.42 0.94 0.11 0.22 0.64 0.56

BMI, body mass index; FFM, fat free mass; WC, waist circumference; HC, hip circumference; WHR, waist to hip ratio; SBP, systolic blood pressure; DBP, diastolic blood pressure; PA, physical activity; BMR, basal metabolic rate; TG, triglyceride; TC, total cholesterol; LDL-c, low-density lipoprotein; HDL, high-density lipoprotein; FBS, fasting blood sugar; HOMA-IR, homeostasis model assessment of insulin resistance

**Table 2:** Clinical and biochemical parameters before and after intervention in the two study groups

Variables	Herbal candy			placebo			p-value ^a^
	Baseline	After 8 weeks	p-value	Baseline	After 8 weeks	p-value	
Weight (kg) BMI (kg/m^2^) Total body fat % FFM (kg) WC (cm) HC (cm) WHR SBP (mmHg) DBP (mmHg) BMR TG (mg/dl) TC (mg/dl) LDL-c (mg/dl) HDL-c (mg/dl) FBS (mg/dl) Fasting insulin (µIU/ml) HOMA-IR	79.8±13.9 31.3±3.8 37±5.9 50.3±10.4 96.8±11.6 111.6±8.4 0.86±0.07 127.4±19.7 80.9±14.9 1687.1±468.9 121.6±38 181.3±44.2 95.1±31.7 45.4±7.2 92.4±14.4 12±9 2.7±2.1	75.8±13.5 29.7±3.8 36.8±6.3 49.3±9.7 92.4±10.9 108.7±8 0.84±0.07 122.9±16.5 79.16±11.1 1599.3±344 102.2±26 176.4±38.9 112.4±35.1 45.7±9 89.5±18.1 9.3±6.8 2.05±1.4	<0.001 <0.001 <0.001 0.01 <0.001 <0.001 0.01 0.11 0.34 0.17 0.01 0.53 0.06 0.90 0.23 0.03 0.03	83.8±15.3 32.5±4.5 38.8±6.2 51.1±10.7 100.3±12.8 114.6±9.4 0.87±0.06 121.9±10.4 78.6±9.7 1854.9±430.1 128±49.9 187.4±38.8 96.2±26.4 42.7±5.4 88.2±8.2 10.8±6.7 2.4±1.6	82.6±15.2 32.1±4.6 38.3±6.3 50.7±10.1 97.2±11.3 112.7±9.4 0.86±0.05 119.8±13.6 76.8±8 1705.7±399.9 110.8±34.9 178.6±39.4 108.3±30.6 45.1±7.1 91.2±8.2 10.5±7.4 2.4±1.8	0.003 0.003 0.10 0.10 0.001 <0.001 0.06 0.31 0.23 0.30 0.06 0.07 0.13 0.12 0.09 0.78 0.94	**<0.001** **<0.001** **0.001** 0.20 0.20 0.14 0.47 0.49 1.00 0.87 0.89 0.67 0.66 0.78 0.052 0.16 0.10

^a^Calculating the difference of each parameter before and after the intervention in each group and compare these changes between the two group; BMI, body mass index; FFM, fat free mass; WC, waist circumference; HC, hip circumference; WHR, waist to hip ratio; SBP, systolic blood pressure; DBP, diastolic blood pressure; PA, physical activity; BMR, basal metabolic rate; TG, triglyceride; TC, total cholesterol; LDL-c, low-density lipoprotein; HDL, high-density lipoprotein; FBS, fasting blood sugar; HOMA-IR, homeostasis model assessment of insulin resistance

**Table:** The effect of the herbal candy and placebo on hunger in breakfast, lunch and dinner meals

Meal		Baseline ^a^	Changes 30 min after herbal candy or placebo ^b^		Changes 1 hour after meal ^c^	Changes 2 hours after meal^ d^		Time effect^ *^			Group effect
										p-value	p-value
Breakfast	Herbal candy	49.9±18.6		-4.2±12.3	-19.7±26		-17.6±29.5		<0.001		0.005
	Placebo	55.2±15.8		-0.6±7.7	-16.5±17.9		-10.2±19.8				
	p-value^#^	-		0.049	0.048		0.004				
Lunch	Herbal candy	58±17.1		-7.2±10.8	-31±17.2		-31.1±17.9		0.04		<0.001
	Placebo	61.7±17.9		-1.2±11.2	-22.1±21.1		-17.8±21.2				
	p-value^#^	-		0.02	0.01		<0.001				
Dinner	Herbal candy	53.3±15.6		-5±10.6	-23.8±19		-25.9±22.4		0.006		0.004
	Placebo	58.3±16.4		-0.3±8.9	-17.8±20.2		-16.6±20.3				
	p-value^#^	-		0.02	0.03		0.005				

*significant difference between a and b, a and c, a and d, b and c, and b and d; ^# ^the rate of reduction of hunger in the herbal candy group compared to the placebo after adjustment for the pre-consumption values as a confounding factor

**Table 4:** The effect of the herbal candy and placebo on fullness in breakfast, lunch and dinner meals

Meal		Baseline ^a^	Changes 30 min after herbalcandy or placebo ^b^	Changes 1 hour after meal ^c^	Changes 2 hours after meal^ d^	Time effect ^*^	Group effect
						p-value	p-value
Breakfast	Herbal candy	40.7±18.4	7.4±10	24.2±22.9	25.1±24.7	<0.001	0.003
	Placebo	42.3±17.4	1.4±10	11.1±20.8	7.7±23.3		
	p-value^#^	-	0.03	0.01	0.003		
Lunch	Herbal candy	38.9±18.8	8.4±11.1	30.9±18.2	31.8±20.1	<0.001	0.004
	Placebo	35.6±18.7	4.6±8.8	20.4±23	17.7±27.3		
	p-value^#^	-	0.10	0.01	0.004		
Dinner	Herbal candy	42.4±17.3	7.9±8	25.6±20.6	27.8±21.6	<0.001	0.01
	Placebo	41.5±19.8	4.2±9.4	15.5±24.8	13.9±29		
	p-value^#^	-	0.09	0.04	0.01		

*significant difference between a and b, a and c, a and d, b and c, and b and d; ^# ^the rate of fullness increase in the herbal candy group compared to the placebo after adjustment for the pre-consumption values as a confounding factor

**Table 5:** The effect of the herbal candy and placebo on eating capacity in breakfast, lunch and dinner meals

Meal		Baseline ^a^	Changes 30 min after herbal candy or placebo ^b^	Changes 1 hour after meal ^c^	Changes 2 hours after meal^ d^	Time effect ^*^	Group effect
						p-value	p-value
Breakfast	Herbal candy	56.3±18.1	-4.6±11.5	-22.3±25.4	-23.6±25.5	<0.001	0.001
	Placebo	58.7±17.8	-1.6±7.9	-11.5±13.6	-8.2±17.2		
	p-value^#^	-	0.10	0.01	<0.001		
Lunch	Herbal candy	59.6±17	-6.4±8.4	-31.6±19.9	-31±20.3	0.02	0.001
	Placebo	63.9±16.6	-1.4±10.4	-17.4±18.2	-14.9±21.8		
	p-value^#^	-	0.01	0.001	<0.001		
Dinner	Herbal candy	55.7±16.4	-5.9±8.8	-25.7±26	-27±23.3	0.04	0.002
	Placebo	61.7±14.5	-2.8±7.5	-15.2±17.4	-15±18.1		
	p-value^#^	-	0.02	0.006	0.002		

*significant difference between a and b, a and c, a and d, b and c, and b and d; ^# ^the rate of reduction of eating capacity in the herbal candy group compared to the placebo after adjustment for the pre-consumption values as a confounding factor

## Discussion

In this clinical trial, we aimed to investigate the effect of a combination of herbal compounds, including peanut oil, *Portulaca oleracea* L., and *Plantago ovata* in the form of a herbal candy on appetite and weight loss in overweight /obese adults. The main findings of the present study suggest that consumption of these herbal compounds can significantly decrease subjective appetite as well as body weight and BMI in overweight/obese adults.

**Effect of peanut and peanut oil on weight and appetite**

Various studies have reported that fats produce a feeling of satiety, and since fat is the predominant source of energy in nuts, whole nuts and their oil can have high satiety properties (Blundell and Rogers, 1991; Kirkmeyer and Mattes, 2000). The effect of nut oil on appetite has been less studied, but nuts are high in unsaturated fats, and studies in rats have shown that unsaturated fats have more satiating effects than saturated fats (Smith, 1998). But studies on humans have not shown convincing results. There are several studies on the effect of oilseeds, including peanuts on weight and appetite (Blundell and Rogers, 1991; Smith, 1998; Kirkmeyer and Mattes, 2000; Coelho et al., 2006; Iyer et al., 2006; Akuamoah-Boateng et al., 2007, Reis et al., 2013).

A randomized cross-over clinical trial by Reis et al. showed that consumption of peanut butter increased peptide YY (PYY), glucagon-like peptide-1 (GLP-1), and cholecystokinin (CCK) concentrations and decreased desire-to-eat in comparison to the control group. The whole peanut led to similar but non-significant effects (Reis et al., 2013). In the present study, it is possible that the increased appetite-suppressing hormones, including YY peptide, caused by the herbal candy, decreased appetite in this group more than the placebo group.

Iayer et al. also examined the effect of peanut oil on appetite; in this clinical trial, the effects of consumption of peanut oil, olive oil, and safflower oil on appetite and food intake during 8 weeks, were investigated. Similar to our study, appetite response was assessed by VAS. Iayer et al. reported that mean ratings of fullness at week 6 and 8 were significantly higher than week 2, while hunger and desire-to-eat ratings did not differ significantly at any time point over the intervention period and no significant differences were detected between treatment groups (Iyer et al., 2006). These findings confirmed the results of our study on the beneficial effects of plant compounds containing peanut oil on appetite control.

In another study, participants received a daily peanut oil dose in a milk-shake equivalent to 30% of their resting energy expenditure for 8 weeks. The study found a significant increase in energy intake and body weight; however, this increase was less than the theoretically predicted weight gain. Coelho et al. also showed no significant within or between-group differences in appetite. It is noteworthy that Coelho et al. had no control group for comparison (Coelho et al., 2006). Also, in the study by Akuamoah-Boateng et al. the effects of peanut oil, olive oil, and safflower oil on energy balance were compared. The results revealed that the average daily energy intake in all 3 experimental groups increased and a significant weight gain was also seen (Akuamoah-Boateng et al., 2007). Contrary to the results of these two studies, in the present study, we revealed that consumption of the herbal candy that contained peanuts for 8 weeks reduced subjective appetite as well as body weight in comparison to the control group. This finding may be due to the combination of peanut oil with other plant compounds that are effective in reducing appetite, including *Portulaca oleracea* and asparagus.

A large proportion of seed oils is comprised of unsaturated fats. These fats are oxidized faster than saturated fats (Jenkins et al., 2008). Although not yet proven in humans, animal studies have shown that oxidized fat leads to a feeling of satiety, while stored fat does not have this effect (Iyer et al., 2006). Therefore, a possible mechanism for the appetite suppressive effect of peanut oil can be due to the effect of the unsaturated fat content of peanut oil. 

In general, the mechanisms of the effects of oilseeds consumption in preventing weight gain or even causing weight loss include:

 1) Satiety: studies have shown that oilseeds contain saturating compounds. The high energy load in oilseeds (such as peanuts, almonds, and hazelnuts that have been studied so far) leads to a severe reduction in the feeling of hunger (Kirkmeyer and Mattes, 2000). Comparison of peanut oil, olive oil, and safflower did not show a difference in satiety; therefore, saturation of fatty acids did not play a role. A low glycemic index of oilseeds has also been suggested as an effective mechanism in reducing appetite (Rajaram and Sabaté, 2006). 

2) Dietary compensation: Consumption of nuts in one meal reduces energy intake in subsequent meals. The fatty acids profile in nuts cannot be responsible for this effect because walnuts, almonds, and peanuts, which have different fatty acid profiles, were found to result in similar effects (about 70-65%) (Alper and Mattes, 2002; Hollis and Mattes, 2007). In addition, dietary compensation in continuous consumption of nuts oil is weaker than consumption of whole nut (about 50-45%) (Iyer et al., 2006). 

3) Efficiency of energy absorption: The efficiency of energy absorption from the nuts is limited (Alper and Mattes, 2002). 

4) Increased energy expenditure: Chronic consumption of nuts can increase energy expenditure, which is not attributed to the thermic effect of food. Evidence shows that the consumption of nuts increases resting energy expenditure (REE) (Alper and Mattes, 2002). Researchers attributed the increase in REE caused by the consumption of nuts to the high content of unsaturated fatty acids and protein in nuts. Because unsaturated fatty acids which are oxidized more than saturated fatty acids and protein, also have a high thermic effect (van Marken Lichtenbelt et al., 1997; Eisenstein et al., 2002). In general, although it is claimed that high-energy foods are problematic for weight loss and maintenance, studies on nuts have proven otherwise. Therefore, nuts can be used to increase the quality and taste of diets without the fear of weight gain (Mattes et al., 2008). 

Recent studies showed that the effect of consumption of oilseeds on the low risk of developing obesity and diabetes can be related to low glycemic index and the high content of fiber, unsaturated fatty acids, and magnesium in oilseeds (Ribeiro et al., 2013). Although nuts are high in calories, studies have shown that their consumption is not associated with weight gain (Alper and Mattes, 2002). The results showed that consumption of oilseeds can increase the feeling of satiety and as a result, reduced calorie intake (Brennan et al., 2010).

**The effect of** ***Portulaca oleracea*** **on weight and appetite**

So far, no study has been done on the effect of *Portulaca oleracea* on appetite, and generally, limited clinical trials exist on the various properties of this plant. In a study, administration of *Portulaca oleracea* powder increased the level of HDL-C and albumin and reduced TC, TG, LDL-C, FBS, insulin, body weight, and BMI in diabetics ( El-Sayed , 2011). Similarly, in the present study, we demonstrated that weight, BMI, and the levels of TG, fasting insulin, and HOMA-IR decreased after the intervention compared to placebo. In an animal trial study by Lan and Fu-er, 2003 consumption of *Portulaca oleracea* significantly reduced weight and serum-free fatty acids and improved insulin sensitivity, glucose tolerance, and lipid metabolism in diabetic rats (Lan and Fu-er, 2003).

Indeed, *Portulaca oleracea* can reduce insulin resistance due to the presence of flavonoids, polyunsaturated fatty acids, and polysaccharides. Additionally, this plant has inhibitory effects on alpha-amylase and alpha-glucosidase, so, it is effective in lowering blood glucose ( El-Sayed, 2011).

**The effect of** ***Plantago ovata*** **on weight and appetite**

Several studies have investigated the effect of different species of *Plantago* major, including Plantago ovata on appetite and weight loss (Hylander and Rössner, 1983; Turnbull and Thomas, 1995; Rigaud et al., 1998; Rodríguez-Morán et al., 1998; Galisteo et al., 2005; Salas-Salvadó et al., 2008). In line with our results, Turnbull et al. demonstrated that Plantago ovata significantly increased fullness one hour after a meal compared to placebo (Turnbull, 1995). Rigaud et al. reported that hunger and energy intake were lower in the intervention group compared to the control group by administration of *Plantago psyllium* (a species of *Plantago* major) (Rigaud et al., 1998).

A possible mechanism underlying the beneficial effects of *Plantago ovata* on appetite and weight loss is that the plant contains soluble fiber that may reduce carbohydrate absorption, postprandial glucose, and insulin secretion. This property is hypothesized to prevent post-absorption hypoglycemia and increase fullness and fat oxidation (Pereira and Ludwig, 2001). Additionally, numerous studies have shown that cholecystokinin levels increase after eating a high-fiber meal. This hormone is secreted by the cells of the small intestine and affects the satiety center and inhibits food intake (Pereira and Ludwig, 2001). Fiber also stimulates the secretion of GLP-1 which slows gastric emptying and leads to appetite suppression (Näslund et al., 1999).

To the best of our knowledge, this was the first study that examined the combined effect of several herbal ingredients including *Portulaca oleracea* on appetite and weight loss based on ITM reference books and modern studies. Another strength of our study was the production of a plant-based product containing herbal compounds effective in weight loss and suppressing appetite, which can be recommended as a safe food product or medicine along with a diet for obese and overweight people. 

We also acknowledge the limitations in our study including 1) lack of measurement for appetite-related hormones before and after the intervention due to financial and time constraints; and 2) inability to accurately standardize the amounts of compounds in the herbal candy. Moreover, due to the limited number of study participants, this does not give us enough strength to generalize the study results to all overweight and obese people. It is crucial to evaluate this preliminary hypothesis by comprehensive and controlled trials studies. However, despite limited clinical findings due to the low sample size, this is the first study that investigated the effect of a combination of herbal mixtures, in the form of a herbal candy on appetite and weight loss.

In conclusion, the findings of the present study demonstrated that a combination of the herbal candy at a dose of 4 g (2 pcs) half an hour before each meal for 8 weeks, could be effective in reducing weight and appetite in overweight and obese people. These results suggest the efficacy of this herbal supplement in controlling and preventing obesity and obesity-related disorders. However, further and larger studies are essential to support these preliminary outcomes. 

## Conflicts of interest

The authors declare that they have no conflict of interest.
